# Rare mosaic variant of *GJA1* in a patient with a neurodevelopmental disorder

**DOI:** 10.1038/s41439-023-00262-9

**Published:** 2024-01-15

**Authors:** Rina Shimomura, Tomoe Yanagishita, Kumiko Ishiguro, Minobu Shichiji, Takatoshi Sato, Keiko Shimojima Yamamoto, Miho Nagata, Yasuki Ishihara, Yohei Miyashita, Keiko Ishigaki, Satoru Nagata, Yoshihiro Asano, Toshiyuki Yamamoto

**Affiliations:** 1 Department of Pediatrics, Tokyo Women’s Medical University, Tokyo, Japan; 2 Division of Gene Medicine, Graduate School of Medical Science, Tokyo Women’s Medical University, Tokyo, Japan; 3 Transfusion Medicine and Cell Processing, Tokyo Women’s Medical University, Tokyo, Japan; 4 Institute of Medical Genetics, Tokyo Women’s Medical University, Tokyo, Japan; 5 Department of Cardiovascular Medicine, Osaka University Graduate School of Medicine, Suita, Japan; 6 Department of Genomic Medicine, National Cerebral and Cardiovascular Center, Suita, Japan

**Keywords:** Neurodevelopmental disorders, Neurodevelopmental disorders

## Abstract

*GJA1* is the causative gene for oculodentodigital dysplasia (ODDD). A novel de novo *GJA1* variant, NM 000165:c263C > T [p.P88L], was identified in a mosaic state in a patient with short stature, seizures, delayed myelination, mild hearing loss, and tooth enamel hypoplasia. Although the patient exhibited severe neurodevelopmental delay, other clinical features of ODDD, including limb anomalies, were mild. This may be due to differences in the mosaic ratios in different organs.

Oculodentodigital dysplasia (ODDD [MIM: 164200]) is characterized by multiple congenital anomalies^1^. The major clinical features include (1) distinctive facial findings, including a thin nose with hypoplastic ala nasi, small anteverted nares, prominent columella, and microcephaly; (2) eye findings, such as microphthalmia and glaucoma; (3) syndactyly, typically digital malformation, including bilateral complete syndactyly of digits 4 and 5 (type III syndactyly), camptodactyly, and permanent joint flexion of the digits; (4) teeth anomalies and enamel hypoplasia; and (5) cardiac dysfunctions^2–4^. Some patients exhibit dysplastic ears and conductive hearing loss^5,6^. Various neurological symptoms, including dysarthria, spastic paraparesis, ataxia, and seizures, occur in ~30% of patients with ODDD^7–10^. ODDD is a genetic disorder inherited as an autosomal dominant trait. In 2003, connexin-43 (gap junction protein alpha 1; *GJA1*) [MIM*121014] on chromosome 6q22 was identified as the gene responsible for this condition^11^. Recently, we identified a novel *GJA1* variant in an undiagnosed patient with a neurodevelopmental disorder; however, the variant was identified as mosaic. The details of this study are as follows.

The patient was a 5-year-old Japanese girl who was the third child of healthy parents. She was born at 37 weeks and 6 days of gestation by vaginal delivery in the cephalic position without any abnormalities. Her birth weight was 2738 g (25–50th centile). Her parents noticed that she showed her eye movements pursuing moving objects but showed no social smiles at 2 months of age. At 12 months of age, she was referred to our hospital because of developmental delay with no acquisition of a sitting position.

On admission, her vital signs were normal, and no cardiac arrhythmias were detected. She displayed a flat nasal bridge and bilateral epicanthus. Very mild webbing was observed between her fingers and toes, except between the first and second fingers and toes. Camptodactyly was also noted in both the fingers and toes. No abnormalities were observed in the skull bone. Neurological examination revealed deep tendon hyperreflexia but no evidence of extrapyramidal signs. Hypotonia or muscle weakness was not observed. An auditory brainstem response revealed mild hearing loss in the left ear. Cranial computed tomography showed bilateral basal ganglia calcifications (Fig. 1a). Brain magnetic resonance imaging revealed mild dilatation of the extracerebral space and lateral ventricles. Delayed myelination of the subcortical white matter and a thin corpus callosum were also observed (Fig. 1b–d).

**Fig. 1:**
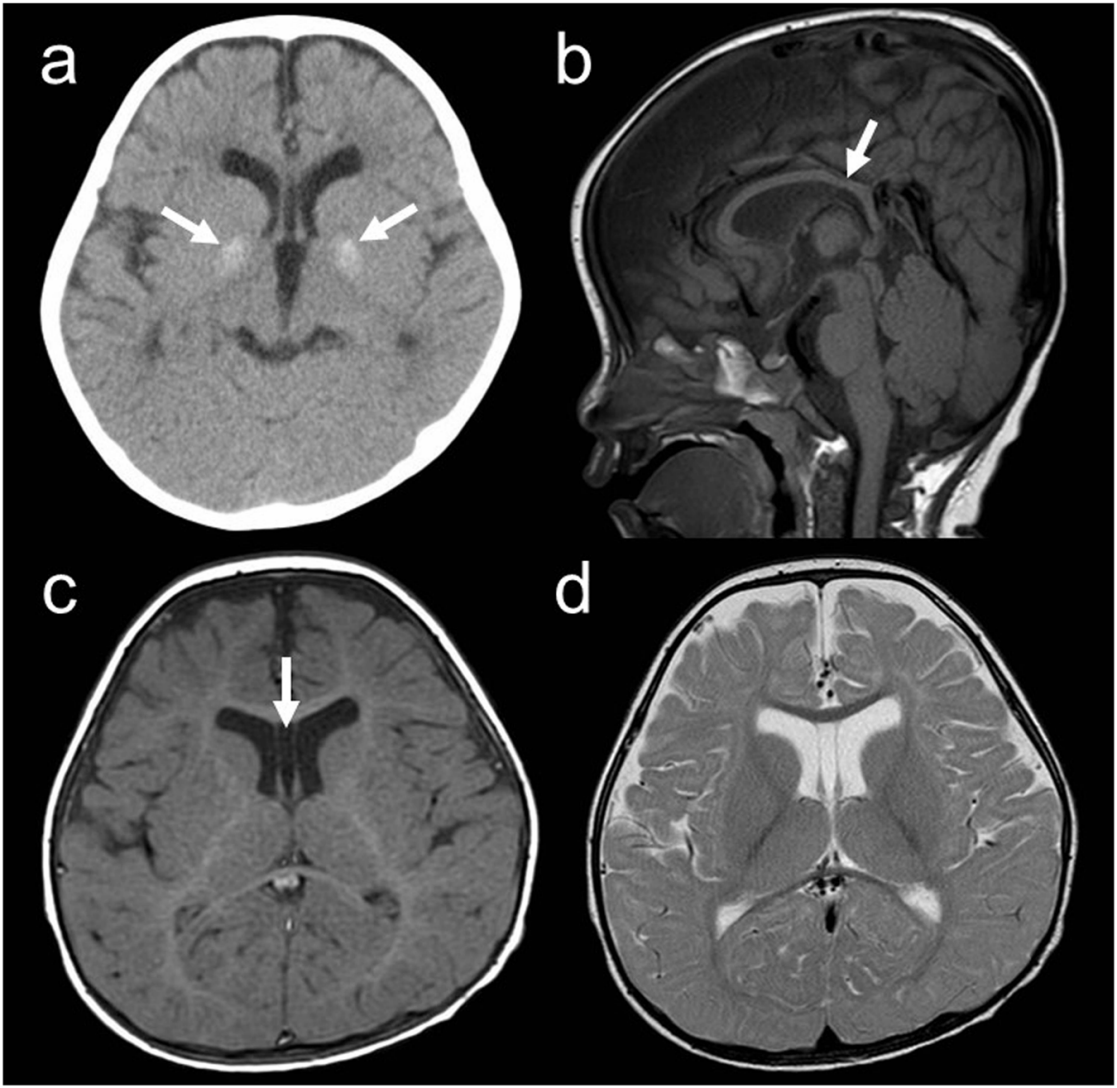
Radiological findings of the present patient. **a** Cranial computed tomography image. Calcification is shown in the bilateral basal ganglia (arrows). **b** Sagittal image of brain magnetic resonance imaging, indicating thinning of the corpus callosum (arrow). **c**, **d** T1- and T2-weighted brain magnetic resonance axial images indicating abnormalities in several regions, including the cavus septum pellucidum (arrow).

At the age of 2 years, the patient experienced her first epileptic seizure. Electroencephalography revealed focal epileptic discharges in the right frontal lobe, and zonisamide was prescribed. She can currently hold a sitting position but has difficulty walking. She does not speak meaningful words but can communicate through babbling and eye contact, indicating severe neurodevelopmental delay. She can eat orally but needs assistance. The erupted lower anterior teeth are fused and have weak enamel. No visual loss or visual field defects were observed. The intelligence scale test showed that her developmental quotient was <20. At present (5 years of age), her height is 97.5 cm (<3rd centile), her weight is 14.1 kg (3rd~10th centile), and her occipitofrontal circumference is 49.5 cm (25–50th centile), indicating short stature.

For precise diagnosis, this patient was enrolled in the research project “Initiative on Rare and Undiagnosed Disorders”^12^, which was performed in accordance with the Declaration of Helsinki and approved by the ethics committee of our institution. After informed consent was obtained from the family, blood samples were collected from the patient and her parents. Genomic DNA was extracted from peripheral blood samples following a standard protocol, and exome sequencing was performed using trio samples, including parental samples, as previously described^13^. GATK HaplotypeCaller was used for variant calling (https://www.broadinstitute.org/). The results revealed a de novo variant of *GJA1* (NM 000165:c263C > T [p.P88L]). This variant is not included in the gnomAD or ClinVar databases. CADD_phred was 28.2, and the MutationTaster_score was 1, suggesting that the variant is damaging. According to the ACMG/AMP guidelines^14^, four scores (PS2, PM2, and PP3) were adaptive. Thus, this variant was classified as “likely pathogenic”.

The identified variant was analyzed using Sanger sequencing. Because there are many homologous genes with similar nucleotide sequences to *GJA1*, PCR primer sets (3′-TTGTCTCTTTGTTTCTTTCAG-5′ and 3′-GTACCACTGGATCAGCAAGAA-5′) were designed at unique sequence sites. For PCR amplification, we used GoTaq® (Promega, Madison, WI). As shown in Fig. 2a, neither of the parents showed p.P88L, and de novo occurrence was confirmed; however, the peak of the minor variant in the patient was lower than that in the wild-type, suggesting the possibility of mosaicism. The read depth of the variant was retrospectively checked, and the read ratio of the minor allele was 8/59 (no contradiction for mosaic status). In this study, GATK HaplotypeCaller was used for variant calling. GATK HaplotypeCaller uses probability calculations to determine whether a variant is heterozygous, homozygous, or wild-type homozygous, even if the proportion of variant reads at depth is less than half. This explains why even a mosaic variant at a low depth could be detected. To confirm the mosaicism ratio, the PCR products were subcloned and inserted into a pGEM-T Vector® (Promega) and transformed into *E. coli*. After transformation, colonies were selected on ABPC-supplemented agar culture medium. The plasmids were extracted from each colony and sequenced. Finally, a minor variant was found in 7 out of 34 colonies (Fig. 2b), indicating that the estimated mosaicism rate in lymphocytes was 41%.

**Fig. 2:**
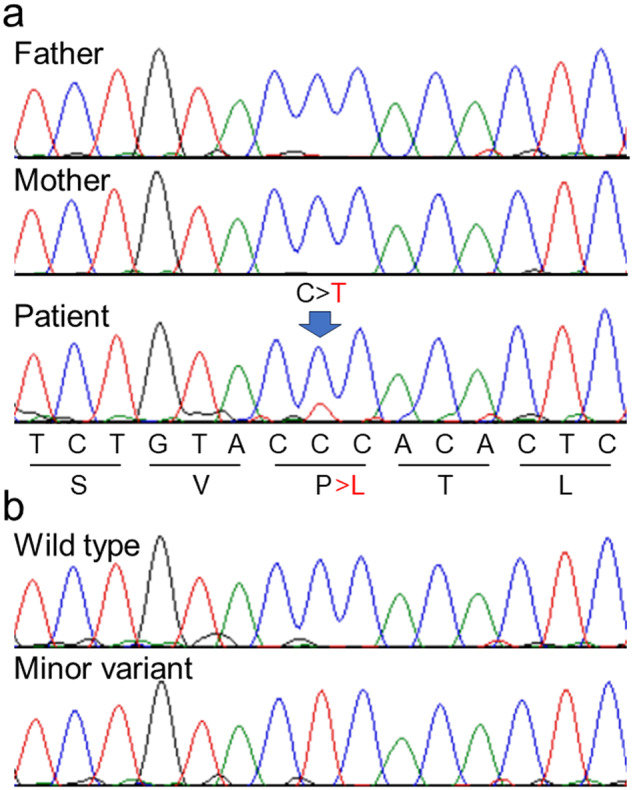
Results of Sanger sequencing. **a** Electropherogram of PCR-direct sequencing showing a very low peak of “T”. **b** After subcloning the PCR products, two types of clones, namely, the wild-type allele and altered minor variant allele, were detected.

Previously, 77 *GJA1* variants have been reported in patients with ODDD or erythrokeratodermia^4,6,15–23^. These findings have been reported in a wide range of medical fields, including pediatrics, dermatology, dentistry, ophthalmology, and orthopedics. This is due to the wide variety of clinical symptoms and severity of this disease, as connexin-43 is pleiotropically expressed in many tissues and regulates intercellular signaling. As shown in Table 1, the present patient exhibited most of the possible clinical findings in ODDD, such as camptodactyly, enamel hypoplasia, hearing loss, and neurological findings. In particular, abnormal brain imaging, including brain calcification, is not rare in patients with ODDD^7,17^. On the other hand, a thin nose and hypoplastic alae, the typical facial findings of ODDD, were not observed. Syndactyly, one of the major findings of ODDD, was also not observed; however, ODDD cases without syndactyly are not rare, and the webbed fingers identified in the present patient were considered to be the consequence of incomplete syndactyly. Therefore, we diagnosed the present patient as having ODDD. Although arrythmia and ocular involvement were not observed, the development of these findings is dependent on the clinical course and may appear in the future. Therefore, careful clinical follow-up is important for this patient.

**Table 1:** Possible findings in ODDD and the present findings in this patient.

	Possible findings in ODDD	This patient
Major findings		
1) Distinctive facial features	Microcephaly	-
	Flat face	+
	Epicanthus	+
	Thin nose	-
	Hypoplastic alae nasi	-
2) Ocular features	Microphthalmia	-
	Glaucoma	-
3) Skeletal features	Syndactyly	Mild (webbing)
	Camptodactyly	+
4) Dental features	Enamel hypoplasia	+
	Others	Fused tooth
5) Cardiac features	Arrhythmia	-
Neurological features		
Developmental delay		+
Epilepsy		+
Brain radiological findings	Delayed myelination	+
	Brain calcification	+
	Cerebral atrophy	+
Other features		
Ears	Mild hearing loss	+
Skin features	Erythrokeratodermia	-

This table is modified from previous tables created by refs. ^4,7^.

GJA1 consists of four transmembrane domains, two extracellular loops, and exposed amino- and carboxyl-termini in the cytoplasm^15^. The p.P88L variant identified in this study is located in one of the transmembrane regions. As shown in Supplementary Fig. 1, most variants in the transmembrane regions are related to ODDD. There were some variants in the region neighboring p.P88L. p.S86Y was identified as a de novo variant in a patient with ODDD^19^ who was first diagnosed with syndactyly and later developed distinctive facial findings and ophthalmological involvement. Psychomotor development was mildly delayed. p.T89I was identified in a familial case of syndactyly with no neurological features^21^. In contrast, the present patient showed severe neurodevelopmental delay but very mild signs of syndactyly (mild webbing of fingers and toes). This may be due to the mosaicism in the present patient. Mosaicism is known to produce atypical or attenuated clinical symptoms due to masking by normal cells^24^. Therefore, we hypothesized that different mosaic ratios in different organs may contribute to the differences in severity of the clinical features in the present patient.

Patients with ODDD who exhibit neurological features as the main finding, but mild syndactyly may not be rare. By applying comprehensive genetic analysis, the existence of such atypical cases has become apparent, and the disease concept is being expanded.

## HGV Database

The relevant data from this Data Report are hosted at the Human Genome Variation Database at 10.6084/m9.figshare.hgv.3354.

## Supplementary information

Supplementary Fig. 1

## Data Availability

The data that support the findings of this study are available from the corresponding author upon reasonable request.
