# Supplementary material for: Rare mosaic variant of GJA1 in a patient with a neurodevelopmental disorder
Source: Hum Genome Var. 2024 Jan 15;11:2. doi: 10.1038/s41439-023-00262-9 (PMC10788341; doi:10.1038/s41439-023-00262-9)
Supplement: Supplementary file 1 — Supplementary Fig. 1 [file 41439_2023_262_MOESM1_ESM.pdf]

Supplementary Figure S1. Schematic representation of the second structure of connexin-43

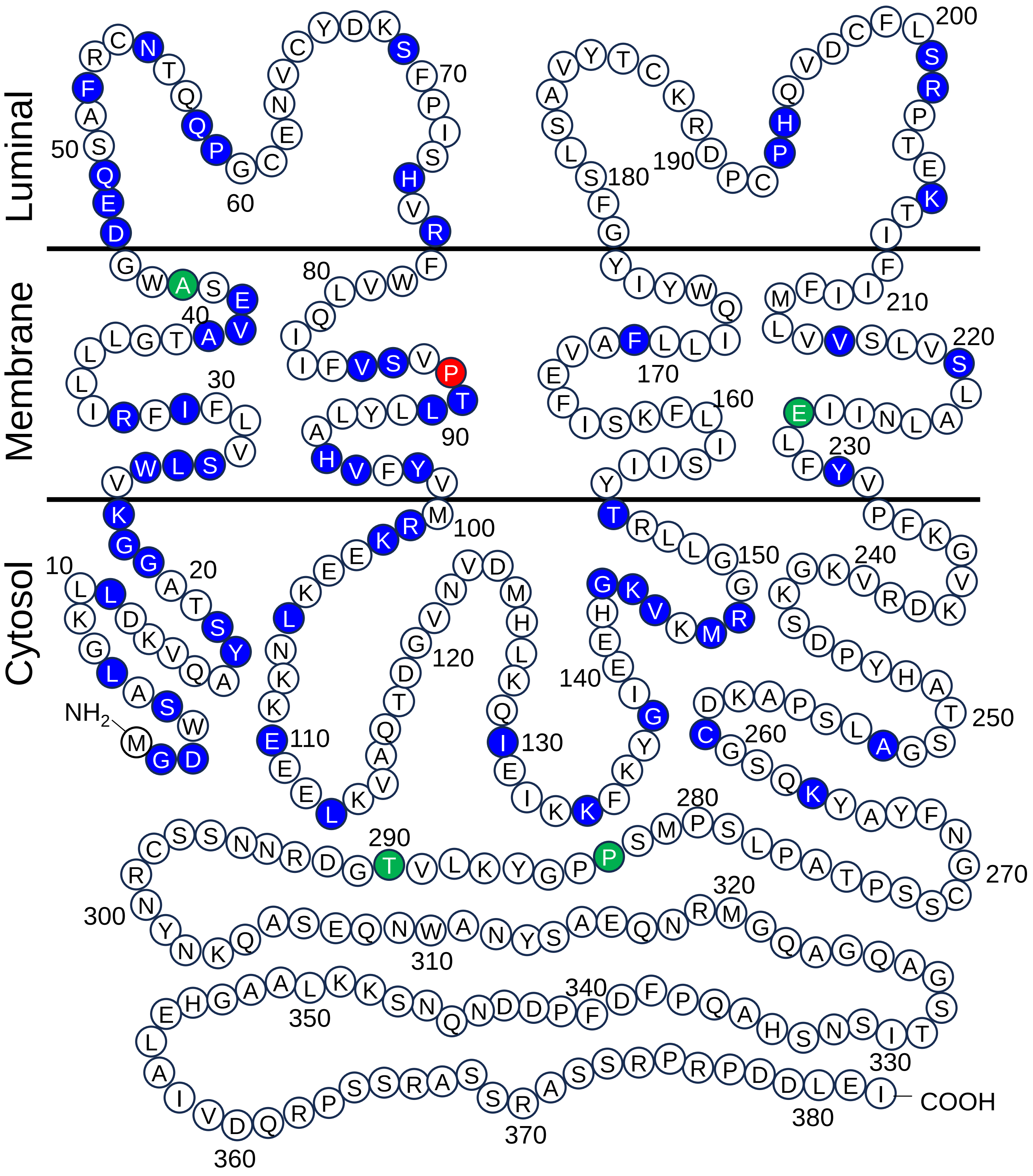

The schema is modified from that reported by Paznekas et al. (2009). This protein has four transmembrane regions. The location of the variant identified in this study is shown by red circle (included in the second transmembrane region). Blue circles indicate the location of the variants found in ODDD cases and green in erythrokeratodermia cases.
